# Editorial: Technologies for neonatal care in LMICs

**DOI:** 10.3389/fped.2024.1462735

**Published:** 2024-09-06

**Authors:** Hippolite O. Amadi, Tina Slusher, Olugbenga Mokuolu, John Ganle

**Affiliations:** ^1^ Department of Bioengineering, Imperial College London, London, United Kingdom; ^2^ Department of Paediatrics, University of Minnesota, St. Paul, MN, United States; ^3^ Department of Paediatrics, University of Ilorin Teaching Hospital, Ilorin, Nigeria; ^4^ Department of Population, Family and Reproductive Health, School of Public Health, University of Ghana, Accra, Ghana

**Keywords:** neonates, LMIC technologies, neonatal care, medical devices, neonatal nursing care, newborn

**Editorial on the Research Topic**
Technologies for neonatal care in LMICs

## 1 Introduction

Newborn babies are among the most vulnerable class of patients in any society. They are entirely incapable of surviving on their own without external help from caregivers and society. A poorly attended newborn will more likely die compared with another who received well-guided and knowledgeable care (1). Therefore, the neonatal mortality rate of any society represents a quick measure of the efficiency of its healthcare system, available technologies, and knowledge base. It is common knowledge that low- and middle-income countries (LMICs) disproportionately contribute over 98% of the global annual burden of neonatal deaths (2, 3). Limited access to sustainable affordable technologies for neonatal care is one of the major impediments in lowering neonatal mortality in LMICs (4). Expensive medical equipment that works well in high-income countries (HICs) may be unsustainable in LMICs due to poor operational infrastructure (5), thus making sophisticated technologies used in HICs unaffordable and unsustainable in LMICs. However, a well-crafted basic technology may be extremely affordable, easily maintainable by in-house technicians, and effective at saving lives.

Therefore, we encouraged researchers to submit their practical demonstrations of applicable LMIC innovations to enable a collection of hybridisable ideas for empowering the rest of the LMICs in neonatal care.

## 2 Outline of contributions

This Research Topic has showcased 10 rigorous studies from 64 collaborating authors across many continents, drawing from easy-to-apply innovative technologies to address a variety of neonatal conditions.

Singh et al. (an Indian–Australian collaboration), explored the “diagnostic utility of lung ultrasound” in predicting when surfactant therapy is needed during neonatal respiratory support. They noted that lung pathologies for respiratory distress at birth have overlapping symptomatology with other conditions, hence the need to research the diagnostic accuracy of a cutoff for the lung ultrasound score (LUS) in predicting the need for surfactant therapy in neonatal respiratory distress. They correlated LUS and the corresponding SPO_2_ to FiO_2_ in 100 neonates and found that an LUS cutoff of 7 predicted the need for the first dose of surfactant.

In another randomised controlled trial, Singh et al. compared the effect of premature infant oral motor intervention (PIOMI) and routine oromotor stimulation (OMS) on oral feeding readiness. They concluded that PIOMI is a more effective oromotor stimulation method for improving oral feeding in preterm neonates.

In a British–Indian collaboration, Hagan et al. assessed the “feasibility of multimodal imaging in neonatal hypoxic-ischaemic encephalopathy (NHIE) from an ovine model.” They argued that the classical “Sarnat staging scale” used in NHIE classification is compounded by difficulties in the clinical detection of seizures. Hence, they proposed a low-cost bedside continuous monitoring electroencephalogram (EEG) tool—functional near-infrared spectroscopy (fNIRS)—that non-invasively measures the electrical activity of the brain from the scalp, capturing the neurovascular coupling (NVC) status. They tested how the imaging system may differentiate between normal, hypoxic, and ictal states in perinatal ovine models. Their main finding was that EEG-fNIRS imaging results are feasible and may provide a biomarker of sepsis effects on the NVC in NHIE.

Rauschendorf et al. (an American–Filipino–British collaboration) presented the effectiveness of a novel bubble continuous positive airway pressure (CPAP) system—Vayu—for neonatal respiratory support in the Philippines, where they compared the clinical outcomes of 1,024 “control” neonates with 979 “test-cases” after the introduction of Vayu bubble Continuous Positive Airway Pressure (bCPAP) systems. They found that Vayu device usage in a neonatal unit resulted in significantly improved outcomes.

Reis et al. —in a Brazilian–Mozambican collaboration—presented a study for enhancing “respiratory distress syndrome (RDS) prediction at birth by optical skin maturity.” They developed a handheld optical device that evaluates the photobiological properties of skin tissue, processing it with other variables to predict early prematurity-related neonatal prognosis and tested the device's ability to predict RDS. The test correctly discriminated RDS newborns with 82.3% accuracy and demonstrated a new way of assessing a newborn's lung maturity, providing potential opportunities for earlier detection and more effective care.

In a pilot preclinical study, Bluhm et al. (USA) demonstrated that their low-cost “self-warming” biomedical device—the NeoWarm—could comparatively assist the hypothermic recovery of six piglets (TEST animal models) when assessed against five other unrecovered piglets (CONTROL) in an induced hypothermia experimental setting. The self-warming “NeoWarm” promises to be potentially applicable—with additional validation—in humans as an alternative to the time-consuming Kangaroo Mother Care technique.

In the first study to identify the factors affecting perinatal and neonatal deaths in Sao Tome and Principe, with collaboration from Portugal, Vasconcelos et al. concluded that the high-risk pregnancy score, meconium-stained fluid, the prolonged rupture of membranes, the transfer from another unit, and instrumental vaginal delivery increased the risk of stillbirth and neonatal deaths by four- to nine-fold. Therefore, prompt intrapartum care is a key strategy that should be implemented in Sao Tome and Principe.

In another fascinating work by researchers from the USA and Cambodia to minimise CPAP-associated oxygen toxicity, Wu et al. conducted a clinical safety assessment of their novel “low-cost entrainment syringe” oxygen blender system for modified bubble CPAP circuits. Thirty-two Cambodian children were included, of which 31 were clinically successful in treatment, as determined by the monitoring of oxygen saturation, the carbon dioxide partial pressure, the fraction of inspired oxygen, the frequency of device adjustments, and the duration of support. The overall outcome declared the blender safe for clinical use.

From the Republic of Korea, Hwang and Lee conducted a cross-sectional study, in which safe-delivery kits were distributed to 534 mothers in rural Ethiopian communities to investigate their impact on preventing newborn and maternal infection. The outcome demonstrates that single-use delivery kits decrease the likelihood of maternal infection, emphasising the need for their adoption in vulnerable countries to improve hygienic birthing, especially for deliveries outside of healthcare facilities.

Finally, a team of Nigerian, British, and Canadian researchers—Amadi et al. —in their courtroom “jury-style systematic review of 32 years of literature without significant mortality reduction,” wondered why high neonatal mortality rates had persisted in Nigeria and some LMICs since the days of “United Nations' Millennium Development Goals (MDG) target (4)”. They reviewed 4,286 publications but only 19 had the potential to reduce neonatal mortality; however, these remained largely unutilised by policymakers. The recommendation from this article was that healthcare systems in LMICs may have to look inwards to strengthen identifiable game-changing discoveries they already possess.

## 3 Concluding remarks

We invite organisations and policymakers of relevant countries to avail themselves of the rich contents of this Research Topic to implement a far-reaching neonatal life-saving campaign across LMICs, inspiring further research for inclusion in our next edition.
